# Effect of small nuclear ribonucleoprotein-associated polypeptide N on the proliferation of medulloblastoma cells

**DOI:** 10.3892/mmr.2015.3148

**Published:** 2015-01-07

**Authors:** JUNJIE JING, YANG ZHAO, CHENGFENG WANG, QINGSHUANG ZHAO, QINCHUAN LIANG, SHOUSEN WANG, JIE MA

**Affiliations:** 1 Department of Neurosurgery, Fuzhou General Hospital of Nanjing Military Region, Fuzhou, Fujian 350025, P.R. China; 2 Department of Pediatric Neurosurgery, The Affiliated Xinhua Hospital of the Medical College, Shanghai Jiaotong University, Shanghai 200092, P.R. China; 3 Department of Pediatrics, Fuzhou General Hospital of Nanjing Military Region, Fuzhou, Fujian 350025, P.R. China

**Keywords:** proliferation, small nuclear ribonucleoprotein-associated polypeptide N, medulloblastoma, short hairpin RNA

## Abstract

Spliceosome mutations have been reported in various types of cancer and a number of antitumor drugs have been observed to tightly bind to spliceosome components. Small nuclear ribonucleoprotein-associated polypeptide N (SNRPN) is a small ribonuclear protein and is a key spliceosome constituent. However, the role of SNRPN in human medulloblastoma remains unknown. In the present study, the effect of SNRPN on cell growth was investigated *in vitro* using the Daoy human medulloblastoma cell line. Lentivirus (Lv)-mediated short hairpin (sh) RNA was used to silence SNRPN expression, which was verified by reverse transcription-quantitative polymerase chain reaction and western blotting. Cell proliferation was examined by MTT and colony formation assays. Knockdown of SNRPN markedly reduced the proliferation and colony formation ability of Daoy medulloblastoma cells. In addition, flow cytometric analysis revealed that the cell cycle distribution was altered when the Daoy cells were infected with Lv-shSNRPN. To the best of our knowledge, this is the first study to investigate the effect of SNRPN on cell proliferation in medulloblastoma. The results indicate that SNRPN may be a potential novel target for the development of pharmacological therapeutics in human medulloblastoma.

## Introduction

Medulloblastoma, an embryonic neuroepithelial tumor of the cerebellum, is the most common type of malignant brain tumor in children and among childhood central nervous system tumors, medulloblastoma has high cancer-associated mortality (1). Medulloblastoma arises from mutated remaining primitive neuroectoderm cells in the ventricle and grows in the cerebellar vermis, regularly invading through the ependyma and brainstem (2). Children with medulloblastoma are generally treated with surgery followed by chemotherapy and radiation, a combination of treatments with five-year survival rates as high as 70–80% (3), but which also induces marked toxicity and adverse cognitive effects that substantially impact the quality of life of the patient.

Medulloblastoma consist of four predominant subgroups with distinct molecular characteristics: Group 1 tumors exhibit mutations in the sonic hedgehog (SHH) gene and its protein receptors; group 2 tumors are induced by changes in WNT signaling, generally through the main responsive gene, β-catenin; group 3 tumors occur due to alterations in transforming growth factor-β-orthodenticle homeobox 2 signaling; and group 4 tumors arise from MYC and MYCN mutations (4–7). For example, SHH signaling pathway mutations are predominantly associated with nodular/desmoplastic and anaplastic medulloblastomas, which presumably arise from granule neuron precursor cells (8). Tumors with activating mutations in the WNT signaling pathway commonly present as the classic medulloblastoma histological subtype, which contributes to 7–15% of medulloblastoma cases (9). However, the majority of oncogenic factors of this heterogeneous cancer remain unknown. Therapies that target key oncogenic promoters, such as SHH, have not been successful, underscoring the importance of identifying alternative targets.

Small nuclear ribonucleoprotein-associated polypeptide N (SNRPN), located on chromosome bands 15q11-13, is imprinted and paternally expressed in somatic tissues and has comparable expression levels in all regions of the brain (10). SNRPN, which is a member of the small nuclear ribonucleic particle SMB/SMN family, is involved in pre-mRNA processing, possibly through tissue-specific alternative splicing events. The SNRPN expression pattern is to a certain extent controlled by methylation in the 5′-untranslated region of exon 1 on the maternally derived chromosome. SNRPN was the first expressed gene identified in the Prader-Willi syndrome (PWS) critically deleted region (10). Previous studies have predominantly focused on SNRPN gene methylation. For example, various studies have demonstrated that SNRPN imprinting may be involved in the regulation of multiple types of cancer, including germ cell tumors (GCTs) (11), acute myeloid leukemia (12) and human uterine leiomyomas (13). SNRPN methylation patterns in germ cell tumors have been reported to reflect primordial germ cell development (11). The variable methylation of SNRPN also supports the hypothesis that intracranial GCTs are associated with neural stem cells (14). In addition, complete maintenance of SNRPN imprinting has been observed in human uterine leiomyomas (13). Analysis of the data from eight papillary thyroid carcinoma (PTC) tumor samples revealed that amplifications of SNRPN occurred solely in tumors with a wild type B-type Raf kinase (BRAF) (15). All the aforementioned results indicate that SNRPN may be critical in tumorigenesis. In the present study, to analyze the role of SNRPN in medulloblastoma, the effect of SNRPN on cell growth was investigated *in vitro* using the Daoy human medulloblastoma cell line.

## Materials and methods

### Cell culture

The Daoy and D283Med human medulloblastoma cell lines were obtained from the Cell Bank of Chinese Academy of Sciences (Shanghai, China). The two types of cells were maintained in Eagle’s minimum essential medium (EMEM) (Sigma-Aldrich, St. Louis, MO, USA) containing 1% L-Glu, supplemented with 10% heat-inactivated fetal bovine serum (FBS) (Sigma-Aldrich) at 37°C in a 5% CO_2_ humidified atmosphere.

### RNA extraction and reverse transcription-quantitative polymerase chain reaction (RT-qPCR)

Total RNA of the cultured cells was extracted using TRIzol^®^ solution (Invitrogen, Carlsbad, CA, USA). RNA quality was assessed with a Bioanalyzer instrument (Agilent Technologies, Palo Alto, CA, USA). cDNA was immediately reverse-transcribed from the isolated RNA using the SuperScript III First-Strand Synthesis system (Invitrogen), and was subsequently used to amplify SNRPN by qPCR using Ex-Taq DNA polymerase (Takara Bio, Inc., Shiga, Japan). Subsequent qPCR amplification was analyzed using the Bio-Rad Connect real-time PCR platform (Bio-Rad Laboratories, Hercules, CA, USA) and was performed using 2 μg cDNA with the following conditions: initial denaturation at 95°C for 1 min, followed by 40 cycles of denaturation at 95°C for 5 sec and annealing extension at 60°C for 20 sec. The absorbance value was read at the extension stage. β-actin served as the input reference. The primers used were as follows: SNRPN forward, 5′-GTTTTGGGTCTGGTGTTGCT-3′ and reverse, 5′-TCATTACCTGCTGGGATGGT-3′; β-actin, forward, 5′-GTGGACATCCGCAAAGAC-3′ and reverse, 5′-AAAGGGTGTAACGCAACTA-3′. The relative mRNA expression levels were determined using the following formula: 2^−ΔCT^ [cycle threshold (CT)], where ΔCT = CT (target gene) − CT (β-actin).

### Construction of SNRPN short hairpin (sh)RNA-expressing lentivirus (Lv)

To produce the SNRPN shRNA-expressing cell lines, an shRNA (5′-AATCTTCATTGGCACCTTTACTCGAGTAAAGGTGCCAATGAAGATTCTTTTT-3′) targeting the human SNRPN gene (NCBI accession number, NM_003097) was inserted into a pFH-L plasmid (Shanghai Hollybio, Shanghai, China). A scrambled siRNA sequence (5′-TTCTCCGAACGTGTCACGT-3′) with no homology to the mammalian genome served as a control (Con). The Lv-based shRNA-expressing vectors were constructed, verified by DNA sequencing, and were designated pFH-L-shSNRPN and pFH-L-shCon. For the transfection, Daoy cells at a density of 5×10^4^ cells/well were seeded in six-well plates and cultured for 72 h to reach 90% confluence. At 2 h prior to transfection, the medium was replaced with serum-free EMEM. The plasmid mixture that contained pFH-L-shSNRPN (or pFH-L-shCon) and pVSVG-I/pCMVΔR8.92 packaging vectors, as well as Lipofectamine 2000 (Invitrogen), was added to the Daoy cells. After 5 h incubation, the medium was replaced with EMEM containing 10% FBS. At 48 h after transfection, lentiviral particles (Lv-shSNRPN or Lv-shCon) were harvested and purified by ultra-centrifugation, according to methods described in previous studies (16,17). At 72 h after infection, the viral titer was determined by counting the number of green fluorescence protein (GFP)-expressing cells under fluorescence microscopy, as described in a previous study (18).

### Western blot analysis

Daoy and D283Med cell lysates were prepared with 2X sodium dodecyl sulfate (SDS) sample buffer containing 100 mM Tris-HCl (pH 6.8), 10 mM ethylenediaminetetraacetic acid, 4% SDS and 10% glycine. The homogenate was subsequently centrifuged at 12,000 × g for 15 min at 4°C and the supernatant was collected and preserved at −80°C. A bicinchoninic acid kit (Pierce, Rockford, IL, USA) was used to determine protein concentration. Protein lysates were electrophoresed on 10% SDS-PAGE gels and then transferred to nitrocellulose membranes using a semi-dry electrotransferring unit (Bio-Rad). The membranes were incubated in 5% non-fat dry milk in tris-buffered saline and Tween^®^ (TBST)buffer containing 10 mM Tris-HCl, pH 8.0, 150 mM NaCl and 0.1% Tween-20 for 2 h at room temperature. Subsequently, the membranes were blotted with the following primary antibodies: Rabbit anti-SNRPN (1:1,000 dilution; #HPA003482; Sigma-Aldrich) or mouse anti-GAPDH (1:1,000 dilution; #sc-32233; Santa Cruz Biotechnology, Inc., Santa Cruz, CA, USA) overnight at 4°C. Following three washes with TBST (5 min each), the blots were incubated with the corresponding horseradish peroxidase-conjugated secondary antibodies: Goat anti-rabbit immunoglobulin G (IgG; 1:5,000 dilution; Santa Cruz Biotechnology, Inc.) and goat anti-mouse IgG (1:5,000 dilution; Santa Cruz Biotechnology, Inc.) for 2 h at room temperature. Immunoreactivity was detected using enhanced chemoluminescent autoradiography (Amersham, Piscataway, NJ, USA).

### MTT assay

Cell proliferation was determined using a MTT colorimetric assay. The MTT assay measures the conversion of MTT to insoluble formazan by the dehydrogenase enzymes of the intact mitochondria of living cells. Subsequent to infection for four days, the Daoy cells were washed with phosphate-buffered saline (PBS), trypsinized with EMEM containing 10% FBS, seeded into a 96-well plate at a concentration of 2.5×10^3^ cells/well and incubated at 37°C. The number of viable cells was measured at daily intervals on days 1–5. At each time-point, 10 μl MTT reagent (Sigma-Aldrich; 5 mg/ml in PBS) was added to the cultured cells and the mixtures were incubated for 4 h at 37°C. At the end of the incubation period, the medium was carefully removed and 100 μl acidified isopropanol containing 10% SDS, 5% isopropanol and 0.01 mol/l HCl was added. The reaction product was quantified by measuring the absorbance at 595 nm using an enzyme-linked immunosorbent assay plate reader (Bio-Rad Laboratories). The experiments were repeated three times.

### Colony formation assay

*In vitro* tumorigenicity was determined on the basis of cell growth in a plate colony formation assay. Daoy cells (500 cells/well) receiving one of three treatments (Con, Lv-shCon or Lv-shSNRPN) were seeded in six-well plates. After six days incubation, the cells were washed with PBS buffer and stained with 0.5% crystal violet in 20% methanol for 20 min. Following crystal purple staining, images of visible colonies were captured and the number of colonies (>50 cells/colony) was counted using Colony Counter software (Image-Pro^®^ Plus version 6.0, Media Cybernetics, Inc., Rockville, MD, USA). The morphology and size of the colonies was examined under a microscope (Olympus Corporation, Tokyo, Japan).

### Fluorescence-activated cell sorting analysis

The Daoy cells were collected five days after infection with Lv-shSNRPN or Lv-shCon, and seeded in 6 cm dishes at 2×10^5^ cells/dish. The cells were then cultured for a further 40 h and subsequently collected. Following washes with ice-cold PBS, the cells were suspended in ~0.5 ml 70% cold alcohol and maintained at 4°C for 30 min. Subsequently, the cells were treated with 100 mg/ml DNase-free RNase (Sigma-Aldrich) and incubated for 30 min at 37°C. Propidium iodide (50 mg/ml; Sigma-Aldrich) was added immediately to the cell suspension. Prior to analysis, the suspension was filtered through a 50 mm nylon mesh and a total of 1×10^4^ stained cells were counted by a flow cytometer (FACSCalibur; BD Biosciences, Franklin Lakes, NJ, USA).

### Statistical analysis

Data were analyzed using GraphPad Prism software version 6.00 for Windows (GraphPad Software, Inc., La Jolla, CA, USA). Values are expressed as the mean ± standard deviation of three independent experiments. The statistical significance of the differences between groups was determined by a repeated-measures analysis of variance test. P<0.05 was considered to indicate a statistically significant difference.

## Results

### SNRPN expression profile in medulloblastoma cell lines

**Figure 1:**
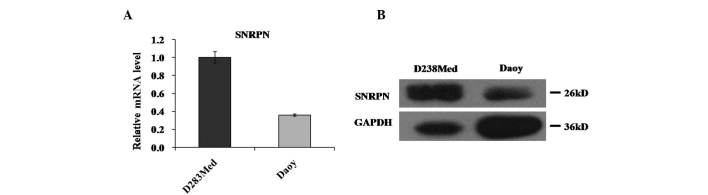
SNRPN expression level profile in Daoy and D283Med human medulloblastoma cell lines. (A) Reverse transcription-quantitative polymerase chain reaction analysis of SNRPN mRNA in the two medulloblastoma cell lines. β-actin served as an internal standard. (B) Western blot analysis of SNRPN in the two medulloblastoma cell lines. GAPDH served as an internal control. SNRPN, small nuclear ribonucleoprotein-associated polypeptide N.

To investigate the role of SNRPN in human medulloblastoma, the expression levels of SNRPN in human medulloblastoma cell lines were assessed. The SNRPN mRNA and protein levels in the two most widely used medulloblastoma cell lines, Daoy and D283Med, were measured. SNRPN mRNA was observed to be highly expressed in the two cell lines, as determined by RT-qPCR (Fig. 1A). Western blot analysis revealed similar results for SNRPN protein, which was highly expressed in the Daoy and D283Med cells (Fig. 1B).

### Knockdown of SNRPN by infection with Lv-shSNRPN in medulloblastoma cells

**Figure 2:**
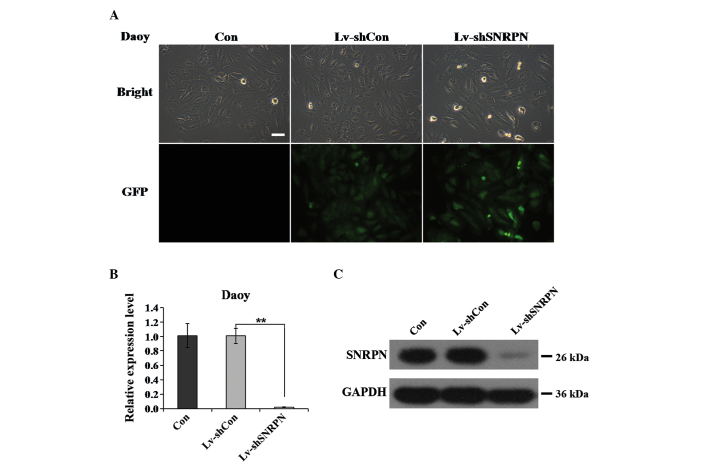
Lv-mediated silencing of SNRPN in Daoy cells. (A) Determination of infection efficiency in Daoy cells. Representative images of Daoy cells after five days of Lv infection are shown (scale bar, 50 μm). (B and C) Expression analysis of SNRPN mRNA and protein levels in Daoy cells following one of three treatments (Con, Lv-shCon or Lv-shSNRPN) by (B) RT-qPCR and (C) western blotting. β-actin gene and GAPDH protein served as internal controls for RT-qPCR and western blotting, respectively. (^**^P<0.01). Lv, lentivirus; SNRPN, small nuclear ribonucleoprotein-associated polypeptide N; RT-qPCR, reverse transcription-polymerase chain reaction; Con, control, sh, short hairpin; GFP, green fluorescence protein.

In order to investigate the role of SNRPN in medulloblastoma, Daoy cells were transfected with Lv that stably expressed SNRPN-specific siRNA (Lv-shSNRPN) and reporter gene GFP. To detect whether the recombinant Lvs successfully infected Daoy cells, GFP signals were observed with a fluorescence microscope (x10 objective lens). The rate of positive enhanced (e)GFP expression in cells remained at >80% subsequent to Lv-shSNRPN and Lv-shCon transfection, which indicated that this lentiviral vector-based RNAi system was successfully established (Fig. 2A).

To further verify that the SNRPN gene was successfully knocked down by Lv-shSNRPN, the expression levels of SNRPN mRNA in the Daoy cells were assessed using RT-qPCR. After three days lentiviral infection, the SNRPN mRNA expression levels in the Daoy cells infected with Lv-shSNRPN were significantly reduced, as compared with the uninfected and Lv-shCon-infected cells (P<0.01; Fig. 2B). The SNRPN knockdown efficiency was satisfactory, with a 98.1% reduction in the Daoy cells infected with Lv-shSNRPN. To confirm the silencing of SNRPN protein, western blotting was performed in the Daoy cells using antibodies against SNRPN. Only a weak band was observed in the Lv-shSNRPN group, although no significant differences in the SNRPN expression levels between the Con group and the Lv-shCon group were identified (Fig. 2C). Therefore, the constructed Lv-shSNRPN effectively knocked down SNRPN expression in the Daoy cells.

### Effect of SNRPN knockdown on medulloblastoma cell proliferation and colony formation

**Figure 3:**
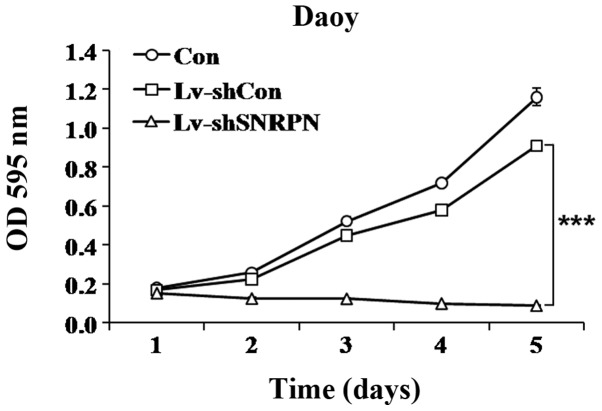
Knockdown of SNRPN inhibited the proliferation of Daoy cells. Cell proliferation in the Lv-shSNRPN groups was significantly inhibited as compared with Lv-shCon-infected and uninfected groups, as determined by MTT assay. (^***^P<0.001). Lv, lentivirus; SNRPN, small nuclear ribonucleoprotein-associated polypeptide N; Con, control, sh, short hairpin; OD, optical density.

To investigate whether the alteration in the levels of SNRPN affected the proliferation of Daoy cells, MTT cell viability and colony formation assays were conducted. A growth curve revealed that the number of Lv-shSNRPN infected cells increased at a markedly slower rate than the uninfected cells or the Lv-shCon infected cells (Fig. 3). On day 5 following Lv-shSNRPN infection, the proliferation rate was markedly reduced, with a 90.2% reduction.

**Figure 4:**
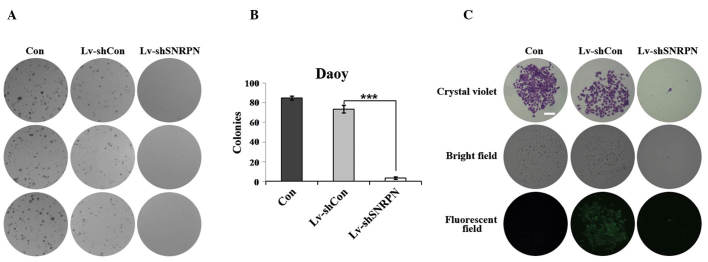
Lv-mediated knockdown of SNRPN inhibited the colony-forming ability of Daoy cells. (A) Representative images of colony formation in Daoy cells with three treatments (Con, Lv-shCon and Lv-shSNRPN) are shown. (B) The size of each colony was significantly smaller in the Lv-shSNRPN groups as compared with the Lv-shCon and uninfected groups (scale bar, 125 μm). (C) The average number of colonies are presented as a bar chart. (^***^P<0.001). Lv, lentivirus; SNRPN, small nuclear ribonucleoprotein-associated polypeptide N; Con, control, sh, short hairpin.

In addition, the Lv-shSNRPN-infected cells produced fewer colonies than the uninfected cells or the Lv-shCon-infected cells (Fig. 4A). Only three SNRPN-depleted Daoy cell colonies were detected as compared with ~85 and 74 uninfected and Lv-shCon infected cell colonies, respectively (Fig. 4B). Furthermore, the size of each colony was also evidently smaller in SNRPN-depleted cells than in the uninfected cells or Lv-shCon-infected cells (Fig. 4C). These data indicate that SNRPN functions as a positive regulator of proliferation and colony formation in Daoy cells.

### Effect of SNRPN knockdown on the cell cycle distribution in the medulloblastoma cells

**Figure 5:**
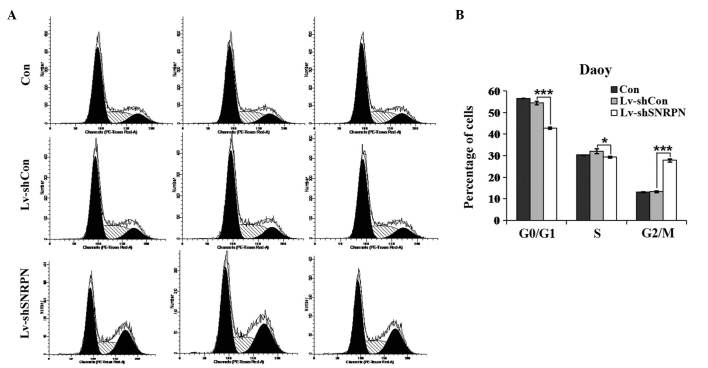
Lv-mediated knockdown of SNRPN induced cell cycle arrest in Daoy cells. (A) Cell cycle distribution of Daoy cells with three treatments (Con, Lv-shCon and Lv-shSNRPN) as determined by flow cytometry. (B) Knockdown of SNRPN in Daoy cells induced cell cycle arrest in the G2/M phase. (^*^P<0.05 and ^***^P<0.001). Lv, lentivirus; SNRPN, small nuclear ribonucleoprotein-associated polypeptide N; Con, control, sh, short hairpin.

To determine the effects of SNRPN depletion on the cell cycle distribution, flow cytometry was conducted on Daoy cells infected with no Lv, Lv-shCon or Lv-shSNRPN, respectively. The proportion of the cell population in G0/G1 phase in the SNRPN-depleted Daoy cells (42.83±0.44%) was significantly lower than those in uninfected cells (56.54±0.12%) and Lv-shCon infected cells (54.47±0.81%), whereas the percentage of the cell population in G2/M phase in the SNRPN-depleted Daoy cells (27.86±0.67%) was markedly higher than those in the uninfected cells (13.12±0.15%) and the Lv-shCon-infected cells (13.39±0.37%; Fig. 5). In addition, the proportion of the cell population in S phase in the SNRPN-depleted Daoy cells (29.31±0.40%) was marginally lower than those in uninfected cells (30.34±0.10%) and Lv-shCon-infected cells (32.14±1.14%). These results indicate that SNRPN may modulate the growth of medulloblastoma cells through the regulation of cell cycle progression.

## Discussion

Medulloblastomas are aggressive primitive neuroectodermal tumors that appear in the cerebellum, Medulloblastomas develop from granule cell-like precursors that have escaped from terminal differentiation. As a histological class, medulloblastoma is the most common type of pediatric brain tumor (19,20). Although there has been marked progress in the understanding of the molecular mechanisms involved in the etiology of medulloblastoma, and in the development of combination treatment using surgery, chemotherapy and radiation, the five-year survival rate for patients with medulloblastoma remains at 70–80%, with the survival rate in young children and infant patients even lower (3). In addition, almost all survivors suffer from adverse, life-long consequences from treatment. These undesirable effects are attributable to the detrimental impacts of surgical procedures, radiation and chemotherapy on the developing brain (21). Therefore, the identification of novel therapeutics that improve the cure rate without harmful side effects is imperative. A previous study demonstrated that medulloblastoma may be divided into four molecular subtypes: WNT, SHH, group 3 and group 4 (7). These four subtypes exhibit notable differences in gene expression, as determined by cDNA microarrays and immunohistochemistry (22,23). Current therapeutic methods that target key oncogenic promoters, such as WNT and SHH, have a number of limitations.

In the present study, SNRPN, a polypeptide of a small nuclear ribonucleoprotein complex, was observed to exert an important role in cell growth in medulloblastoma cell lines. Knockdown of SNRPN by infection with SNRPN-specific siRNA markedly inhibited the proliferation of the medulloblastoma cells. In addition, a colony formation assay was used to assess the capability of cell growth in anchorage-independent conditions comparable with the *in vivo* situation (24). Knockdown of SNRPN may also impair the anchorage-independent growth of medulloblastoma cells. Altogether, to the best of our knowledge, this is the first study to report that SNRPN exerts a key role in medulloblastoma cell growth.

To further investigate the mechanism of cell growth inhibition, the cell cycle progression of the Daoy medulloblastoma cells was determined by flow cytometric analysis. The results revealed that the cell cycle distribution was altered following Lv-shSNRPN infection. SNRPN knockdown significantly increased the percentage of cells in G2/M phase, while simultaneously reducing the proportion of cells in G0/G1 phase. The results raise the question of the underlying mechanism of SNRPN regulation of the medulloblastoma cell cycle. SNRPN is involved in alternative splicing of pre-mRNA, possibly in a tissue-specific manner (25). We hypothesize that SNRPN recognizes specific nucleic acid sequences of specific genes involved in the G2/M and the G1 cell cycle checkpoints, such as p53 and p21 (26,27) and regulates the expression of the corresponding protein molecules. However, due to the limitation of the present study, the specific underlying mechanism was not further investigated.

SNRPN was the first protein in the PWS critically deleted region to be identified. Numerous studies concerning the methylation of the SNRPN gene in cancer have been published. For example, SNRPN methylation patterns in germ cell tumors have been reported to reflect primordial germ cell development (11). The variable methylation of SNRPN also indicates an association between intracranial GCTs and neural stem cells (14). In addition, complete maintenance of SNRPN imprinting has been observed in human uterine leiomyomas (13). Analysis of the data from eight PTC tumor samples revealed that amplifications of SNRPN occurred solely in tumors with a wild-type BRAF (15). According to the results of previous studies and those of the present study, SNRPN regulates various tumor characteristics, including proliferation, colony formation and the cell cycle; therefore SNRPN methylation in medulloblastoma may also be altered and may be associated with a specific subtype of medulloblastoma. The SNRPN methylation level in medulloblastoma requires further analysis.

In conclusion, to the best of our knowledge, this is the first study to define SNRPN as a functional mediator of medulloblastoma cell growth. Knockdown of SNRPN was demonstrated to significantly inhibit medulloblastoma cell growth and induce G2/M phase arrest *in vitro*. Thus, the present study may provide a novel therapeutic approach to treat patients with medulloblastoma.
